# Adapting an m-Health Intervention for Spanish-Speaking Latinx People Living with HIV in the Nonurban Southern United States

**DOI:** 10.1089/tmr.2020.0018

**Published:** 2021-02-03

**Authors:** Tabor E. Flickinger, Jacqueline E. Sherbuk, Kristen Petros de Guex, Diego Añazco Villarreal, Michelle Hilgart, Kathleen A. McManus, Karen Ingersoll, Rebecca Dillingham

**Affiliations:** ^1^ University of Virginia Department of Medicine, Division of General Medicine, Geriatrics, and Palliative Care, Charlottesville, Virginia, USA.; ^2^ University of Virginia Department of Medicine, Division of Infectious Disease and International Health, Charlottesville, Virginia, USA.; ^3^ Colegio de Ciencias de la Salud, Escuela de Medicina, Universidad San Francisco de Quito, Quito, Ecuador.; ^4^ University of Virginia Center for Behavioral Health and Technology, Charlottesville, Virginia, USA.; ^5^ University of Virginia Department of Psychiatry and Neurobehavioral Sciences, Charlottesville, VA, USA.

**Keywords:** HIV, intervention, Latinx, m-health, peer support, Spanish

## Abstract

**Background:** Latinx people in the United States are disproportionately diagnosed with HIV and are more likely to experience worse HIV-related health outcomes. Although m-health has demonstrated success in improving HIV care, a gap remains in the development of m-health platforms tailored to Latinx populations.

**Methods:** We conducted formative study to guide the adaptation of an evidence-based m-health intervention, PositiveLinks (PL), for Spanish-speaking Latinx people living with HIV (PLWH). Spanish-speaking Latinx PLWH in the nonurban Southern United States completed semistructured interviews and viewed a demo version of the m-health intervention. Qualitative analysis was performed using a grounded theory approach. Emerging themes were identified in four topic areas: (1) prior experiences with technology, (2) desired m-health features, (3) experiences with prototype app, and (4) iteration of prototype.

**Results:** All PLWH who participated (*n* = 22) were born outside the continental United States. Participants included 10 men, 10 women, and 2 transgender participants. Mean age was 41.1 years (standard deviation 11.6 years). Participants expressed concerns about privacy, a need for reliable information, and interest in practical m-health features such as appointment and medication reminders. After trialing the Spanish-language PL prototype, participants reported that peer support and positive reinforcement were strong motivators to use the app. The ability to individualize the app to meet one's own needs was also considered important.

**Conclusion:** This formative study provides baseline attitudes about m-health among Latinx PLWH as well as desired m-health features. m-Health interventions are acceptable to Spanish-speaking PLWH and involving the target population in a user-centered formative process led to improvements in app accessibility and usability.

## Introduction

The Latinx population in the United States is disproportionately affected by HIV. New HIV diagnoses continue to rise among Latinxs, despite an overall decrease in new diagnoses across the United States.^1,2^ Key Latinx populations impacted by HIV at high rates include men who have sex with men, transgender women, and people born outside the United States.^2,3^ Latinx people living with HIV (PLWH) face disparities in outcomes at many stages along the HIV care continuum, and it is estimated that only 51% of Latinx PLWH are virally suppressed.^4^ Latinx PLWH face numerous barriers to care, including language, cultural differences, immigration status, and limited access to insurance.^3,5,6^ An identified gap in the care of Latinx PLWH in the United States is the need for evidence-based interventions to improve engagement in HIV care that are tailored to Latinx communities.^3^ m-Health may provide a solution to fill this gap.^7,8^

Mobile technology interventions have expanded greatly in recent years, targeting a variety of medical conditions and health behaviors. These interventions include texting (short message service-based) strategies to send reminders or messages, online portals, and applications (apps) with interactive functionality. Apps can help users track health-related data, goal-setting, access information and resources, and communicate with peers or with health care providers. A growing body of evidence indicates that m-health interventions can make an impact on health outcomes, including diabetes, mental health, chronic lung disease, and cardiovascular disease.^9,10^ Recent studies also suggest that formative study with target users and strategies of user-centered design can improve the usability of apps.^11–13^ In the United States, most m-health interventions have been developed for English-speaking populations. App development for non-English speakers has been primarily focused on medical translation programs to address limited access to interpreters.^14^ There is an unmet need for m-health interventions addressing the needs of non-English speakers managing chronic health conditions, including HIV.

In the nonurban South, a low density of Spanish-speaking PLWH and a paucity of Spanish-speaking health care providers leads to a lack of social support among peers and difficulty accessing health care services.^6^ PositiveLinks (PL), an evidence-based m-health intervention for PLWH,^15^ is a clinic-based m-health platform that includes daily tracking of medication adherence, mood and stress; appointment reminders; educational resources; laboratory results; and secure messaging with clinic staff and providers (www.positivelinks4ric.com). PL was developed at and is owned by the University of Virginia where continued research and development occurs. PL also provides access to a private community message board that creates an anonymous environment for PLWH to discuss self-selected issues. PL improves long-term engagement in care and viral suppression among a general sample of PLWH at a university-affiliated Ryan White clinic.^16^ PL was developed based on best practices for chronic disease management^17^ and user-centered design.^18^ The developers employ benchmarking as a summative method of evaluating user experience by collecting analytic, quantitative, and usability feedback from PLWH who use the program, to make ongoing improvements to the PL platform and features.^19^ However, preferences of Spanish-speaking Latinx PLWH may differ from those of English-speaking PLWH with whom the app was originally developed, and tailoring existing m-health interventions to Latinx PLWH has been identified as a need in the literature.^20^ Culturally tailored health care requires input from patients^21^; therefore, we conducted formative study to guide adaptation of PL for Spanish-speaking Latinx PLWH.

## Methods

### User-centered design framework

The concept of user-centered design describes processes in which the end users have some influence over the design of a system or product.^18^ This methodology can be manifested in involving users at key phases, from needs assessment to initial design to iteration of prototypes. In m-health, this approach includes users in providing input on desired features and functionality, so that their input can be incorporated into the development process with the goal of improving usability and the intended outcomes of interventions.^22^ We incorporated principle of user-centered design in this study by involving Spanish-speaking Latinx PLWH in iterative formative study to adapt PL to meet their needs.

### Qualitative research method and grounded theory

m-Health development processes that seek user input generally rely on qualitative methods to gather and analyze data. Focus groups are commonly used to bring potential users together for brainstorming and discussion. Individual interviews can also be used, especially in situations where privacy concerns may complicate focus group formation, as in our study of PLWH. For our methodology, we selected a grounded theory approach, which emphasizes the application of inductive techniques to elicit emerging themes.^23^ This approach was chosen to focus on participants' experiences and discern relevant themes of importance to members of our target population.

### Study design and population

Participants were Spanish-speaking Latinxs age >18 years. The inclusion criteria required for participation were age ≥18 years, known diagnosis of HIV, native Spanish language fluency, and ability to provide informed consent. Exclusion criteria was inability to use the app due to severe cognitive or physical impairment. Participants could be bilingual in English and Spanish. Participants were recruited by telephone and in person from two settings: (1) a nonurban Ryan White HIV/AIDS Program (RWHAP) clinic located in the Southern United States, and (2) a Latinx-serving community-based organization (CBO). The RWHAP clinic serves ∼850 clients from 52 primarily rural counties in western Virginia. Approximately 5% of clients self-identify as Hispanic. The CBO is in the District of Columbia metropolitan region and serves primarily clients who speak Spanish. Potential participants were identified as those RWHAP clinic patients documented to prefer to receive care in Spanish and patients identified to be Spanish speaking at the CBO. Convenience sampling was used to enroll participants. Interviews took place between January 2019 and June 2019. This study was reviewed and approved by the University of Virginia Institutional Review Board for Health Sciences Research. Informed consent, conducted in Spanish, was obtained from all participants.

**FIG. 1.**
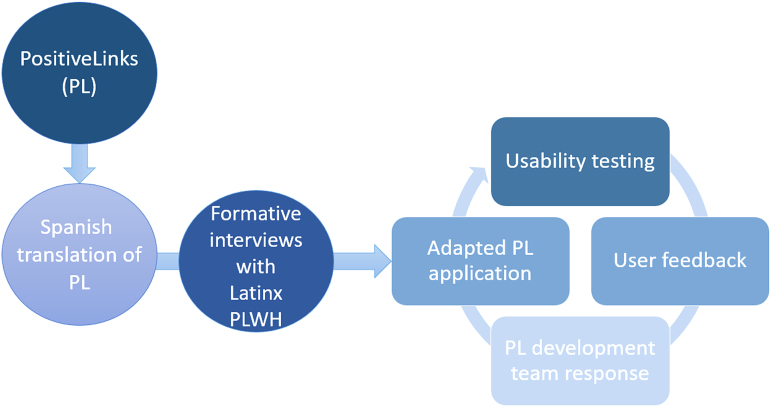
Process diagram for adaptation of PositiveLinks, an m-health application, to Spanish language, incorporating feedback from Spanish-speaking PLWH. PLWH, people living with HIV.

The existing English-language PL app was translated to create a Spanish-language prototype that could be shown to study participants. All participants completed a semistructured interview. Information obtained through qualitative interviews was used to modify the app in an ongoing iterative process (Fig. 1). Participants who raised concerns with app features were invited back for a second interview 6–9 months later to evaluate a revised version.

### Data collection

Interviews were conducted in person in the clinic or at the CBO where participants were recruited. The interviewers were two bilingual Spanish- and English-speaking study personnel, one a medical student and the other with a graduate degree in Latin American studies and background studies in anthropology and social sciences. Interviews were audiotaped, transcribed, and translated. Translations were performed by two study team members, one a native Spanish speaker and the other a native English speaker with national medical interpretation certification. All translations were reviewed by both team members and discrepancies resolved by returning to the original audio file.

Semistructured interviews were designed to explore (1) participants' attitudes toward technology, (2) participants' desired m-health features, and (3) feedback on a Spanish-language PL prototype (Supplementary Table S1). Interviews ranged from 20 to 65 min in length and participants received a gift card as compensation. Some participants gave additional feedback on an iteration of the Spanish-language PL app with modified features based on their earlier feedback. Participants were queried about demographic characteristics, including self-reported English proficiency, and asked to respond to open-ended questions about their experiences living with HIV and their prior experiences and attitudes toward technology. The interviewer then demonstrated the Spanish-language PL prototype and asked for the participants' impressions of its functionality and its linguistic and cultural acceptability. Participants who expressed dissatisfaction with the laboratory results feature of PL were invited to engage in a follow-up usability interview after the feature was modified in efforts to enhance its user-friendliness and accessibility.

### Analysis

Interviews were imported into Dedoose for analysis (Dedoose Version 8.0.35, web application for managing, analyzing, and presenting qualitative and mixed method research data [2018]. SocioCultural Research Consultants, LLC, Los Angeles, CA; www.dedoose.com). The initial codebook was developed using principles of grounded theory, then refined iteratively. Each interview was coded by at least two team members with discrepancies resolved by consensus. Data saturation was achieved after analysis of 18 interviews, with no additional codes added for the subsequent interviews. The final codebook was applied to all 22 interviews. Thematic analysis was performed to identify key aspects of participants' experiences and preferences for m-health that may be relevant to intervention tailoring.

## Results

### Participant characteristics

A total of 28 people were approached and invited to participate with 22 enrolling for a participation rate of 79%. Six candidates who were contacted declined to participate in the research, citing transportation, work responsibilities, and confidentiality concerns as barriers to their participation. Participants included 10 men, 10 women, and 2 transgender women (Table 1). Mean age was 41.1 (standard deviation [SD] 11.6) years. All participants were born outside the continental United States, with the most prevalent countries of origin being Honduras (*n* = 7), Mexico (*n* = 4), and El Salvador (*n* = 4). Participants had lived in the continental United States for a mean of 12.2 (SD 9.8) years with a range of 0.5–45.0 years. Most participants reported limited or no English proficiency (*n* = 14, 64%) and spoke only Spanish at home (*n* = 20, 91%).

**Table 1.** Participant Characteristics (*N* = 22)

Characteristic	*N* (%)
Gender	
Male	10 (45)
Female	10 (45)
Transgender	2 (9)
Age group	
18–29 years	3 (14)
30–39 years	7 (32)
40–49 years	6 (27)
50–59 years	4 (18)
60+ years	2 (9)
Region of origin	
Central America (Honduras, Mexico, El Salvador, and Guatemala)	17 (77)
South America (Venezuela, Peru, and Argentina)	3 (14)
Caribbean (Dominican Republic and Puerto Rico)	2 (9)
English proficiency	
None	9 (41)
Limited	5 (23)
Basic	4 (18)
Advanced	4 (18)
Language spoken at home	
Spanish only	20 (91)
English only	2 (9)

### Qualitative analysis

Qualitative analysis was performed using grounded theory, as described in the Methods section. Emerging themes were identified in four topic areas (Table 2).

**Table 2.** Major Topics Addressed During Participant Interviews and Emerging Themes from Qualitative Analysis

Topic	Emerging themes
Prior experiences with technology	1. General use of technology has negative associations, especially related to risks of unreliable information and concerns about privacy
	2. Technology use is limited by structural and individual barriers
	3. Positive aspects of technology are those aspects related to personal connection
Desired m-health features	1. Practical features—access to reliable and trustworthy HIV-related information, appointment reminders, and medication reminders
	2. Interpersonal aspects of m-health—support, care connection, and social connection were desired by some participants
Reaction to prototype app	1. Nearly all participants were motivated to use the app and felt that the app was linguistically and culturally appropriate
	2. Access to information was regarded to be of high interest
	3. Positive reinforcement/support among peers and care connection were strong sources of motivation to use the app after seeing examples of potential interactions
	4. Individualization of the app was desired to meet particular needs
Reaction to iterative prototype	1. Features revised based on participants' feedback were felt to be improved

#### Prior experiences with technology

Participants expressed positive and negative prior experiences with technology. One of the most common positive associations with technology was personal connection (*n* = 20, 91%). Participants used social media tools, such as WhatsApp, to keep in touch with friends and family in their country of origin. Access to information was another commonly cited use for technology (*n* = 15, 68%), although some participants also expressed a desire for more information in Spanish and relevant to the Latinx community (*n* = 6, 27%). Only a few participants had used technology to seek positive reinforcement/support (*n* = 4, 18%) or connection to their health care (*n* = 3, 14%).

Prior negative experience with technology was described by 16 participants (73%). The most common negative experience described was difficulty using technology and low confidence with use, expressed by nine participants (41%). As one participant stated, “It's hard for me to use computers for those things. I haven't been able to use it well.” Individual and structural barriers were present, including lack of understanding, low literacy, poor vision, and poor internet connection. Eight participants (36%) also expressed concern about unreliable or conflicting information found online: “What did not help were the discussion forums because you'd hear too many things. So, you don't know if everything is real or not, and that makes you doubt.” Five participants (23%) had experienced negative consequences of technology use, including loss of privacy: “You can't do something privately because, all of a sudden, someone else hears about it. So, like, it's not very secure.”

#### Desired m-health features

When asked what they wished for in an m-health program for HIV care, most participants prioritized access to information (*n* = 17, 77%), appointment reminders (*n* = 15, 68%), and medication reminders (*n* = 12, 55%). Participants discussed the importance of accurate information about HIV: “Before I was infected, well, to me, HIV meant that you're going to die soon. And now that I'm positive, well, now I have other ideas about HIV … So, that's where the application would come in. Because later, you would start researching and all that.” App-based reminders were cited as an important component of managing one's HIV care: “Sometimes we're involved in a thousand different things and we forget, so, a reminder would be very good, too.” Although not mentioned by as many participants, other themes related to living with HIV were also regarded as important, including positive reinforcement/support (*n* = 9, 41%), care connection (*n* = 7, 32%), social connection (*n* = 6, 27%), mental health resources (*n* = 6, 27%), and linguistic/cultural relevance (*n* = 6, 27%).

#### Experiences with PL prototype

After discussing prior experiences with technology and m-health preferences in general, the interviewers demonstrated the Spanish-language PL prototype. After viewing the prototype, most participants (91%) felt they would be motivated to use the app: “My health is important and it's also important to expand my support network. So, for those reasons, I see it as motivating.” Aspects of PL highlighted commonly by participants included access to information (*n* = 19, 86%), positive reinforcement/support (*n* = 18, 82%), personal connection (*n* = 14, 64%), ease of use (*n* = 10, 45%), privacy (*n* = 10, 45%), and connection to their care team (*n* = 9, 41%).

Positive reinforcement/support had not been as frequently mentioned as a desired feature before viewing the prototype; however, observing the PL community message board allowed participants to envision its use. One participant stated, “We all have times when we feel discouraged, and maybe seeking out positive comments from other people who are in the same situation as we are,” would be helpful. Others perceived engagement with a virtual community of people living well with HIV as a source of hope and motivation: “A person who lives this way, there are many lessons for you. There are ways to control the disease, ways to live positively, keeping up with your appointments, with your medications—that's why it's here. For me, it's magnificent.” Personal connection was also seen as an important aspect of the community board: “Yes, to converse, without feeling ashamed. Yes, there are times when you're stuck in the same routine, and, well, conversing with other people, you can make new friends and everything.”

Care connection was also viewed positively by participants. The secure messaging feature provides a means of reaching out to the HIV clinical team. In one participant's words, “It's like being connected with care; everything from doctors, if you feel bad, say—if you want to make an appointment with your psychologist, with your nutritionist, with your doctor—you can send a message directly. Or sometimes in the clinic, there are times when nobody answers; you can send a message to your doctor.”

Although most participants reported the prototype was linguistically and culturally appropriate (*n* = 19, 86%), some expressed difficulty understanding certain features. Specifically, the laboratory results section, where CD4 count and HIV viral load are presented, was unclear. Some features did not fit participants' individual needs. For example, some reported that they already had good adherence to their antiretroviral therapy and would not need reminders for taking medication. Others did not think they would make use of the community board, due to feeling uncomfortable sharing information with people they did not know.

#### Iteration of PL prototype

Most participants (*n* = 16, 73%) suggested potential improvements during the initial interview. Priorities included creation of an integrated Spanish-language community board to bring together different clinical sites, rather than the demonstrated board restricted to a single clinical site, and redesign of the laboratory results feature. Participant suggestions that led to changes in the revised app are noted in Table 3.

**Table 3.** Modifications to the Spanish PositiveLinks Application Based on Examples of Participant Comments or Suggestions for Improvement

Category	Participant comments	Modifications
Overarching themes		
Linguistic and cultural relevance	• PL staff posts and announcements should be in Spanish to be more inclusive of limited English proficient used and to encourage their participation.	• The Spanish language app has launched. All PL staff posts and announcements are now in Spanish.
	• Spanish-speaking users should be able to switch between English and Spanish community message boards so the communities are not segregated by language preference, especially as some users are bilingual.	• A Spanish language community message board was created that is separate from the English language board, but Spanish speakers also have access to the English board.
Accessibility	• Include provider photographs and phone numbers next to their names in the contacts section to accommodate low literacy levels.	• Provider photographs have been added to the contacts page.
	• Video introduction to the app	• A video introduction to PL in Spanish is now available on the app.
	• Access to a point of contact for issues that arise with the app	• A contact person is available in person daily in the clinic.
Communication	• Ability to ask questions and receive answers in real time	• The community message board now has a Q and A section to facilitate this.
	• Space to ask private questions to specialists in clinic	• Private messaging with providers is available.
Community	• In-person social events to build community and develop a deeper understanding of others’ lives and experiences	• Online drop-in sessions have been developed through an embedded telehealth feature.
Wellness	• More features related to emotional health	• The weekly quiz on the app now includes questions related to emotional health.
	• More information about healthy living with HIV and other medical concerns	• Providing education about different types of wellness through weekly quizzes
	• Surveys for self-reflection on emotional health, wellness, and physical well-being should be provided.	• A polling feature was implemented to allow for reflection and feedback about the community's perspectives.
Suggestions for specific app features		
Laboratory results	• The results chart for CD4 and viral load is not user-friendly and is difficult to understand. It should include accessible language and more information about what desirable CD4 and viral load ranges are.	• The results feature was completely redesigned based on this feedback.
Reminders	• Alarm to remind users of medical appointments two days in advance.	• An appointment reminder has been added to the app.
	• Customize the daily medication reminder to a time convenient for the user.	• Users can now customize the timing of the medication reminder.
Resources	• Updates regarding medical or research advances in HIV should be available on the app.	• A new process was developed for members to suggest new resources for inclusion in PL and has been used frequently by members.
	• App users should be able to suggest relevant resources that they think would benefit other PLWH to PL staff.	
	• Information on varied topics including parenting as a PLWH, U = U, immigration status, and combatting stigma	

PL, PositiveLinks; PLWH, people living with HIV; U = U, undetectable equals untransmittable.

**FIG. 2.**
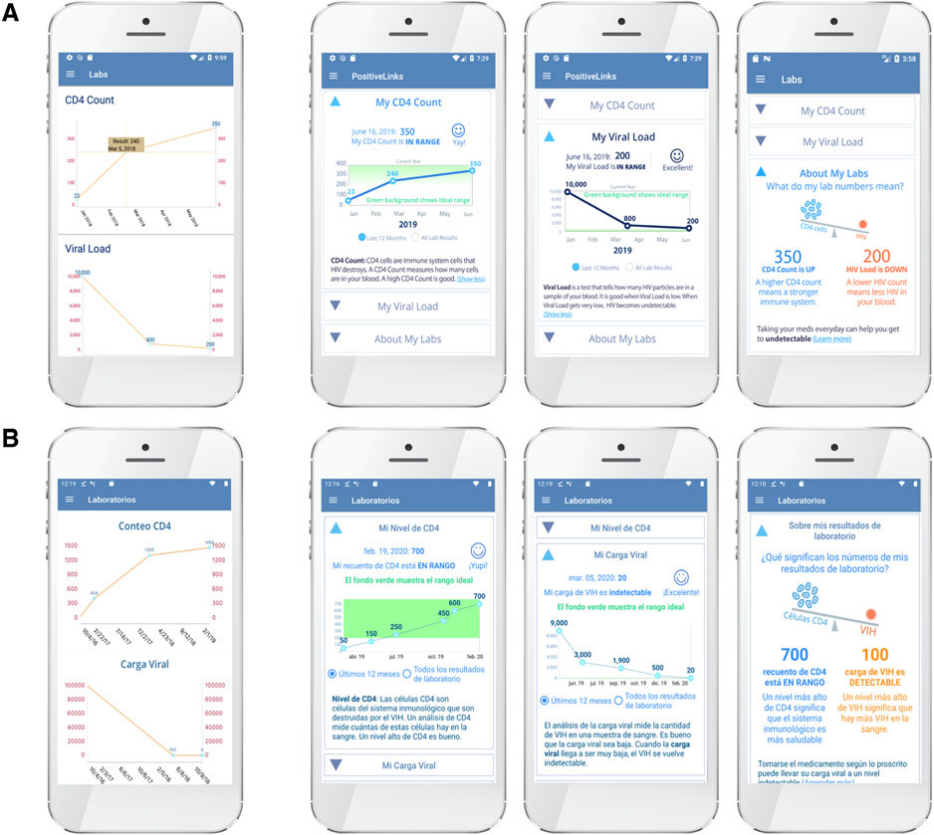
PositiveLinks laboratory results feature redesign. **(A)** English and **(B)** Spanish. The image on the left demonstrates the original design, which participant feedback identified to be confusing and did not improve their understanding of their health. The three images on the right demonstrate the redesigned laboratory results feature incorporating participant input.

Three participants who expressed difficulty with the laboratory results feature stated that the graph was challenging to interpret relative to their HIV-related health status. All three participants who voiced concern with the laboratory results feature had follow-up interviews to elicit feedback on the redesigned display. All three reported improved understanding of the visuals and text. The redesigned laboratory results feature includes definitions of CD4 count and viral load in accessible lay language; color-coded graphs identifying optimal ranges for laboratory values with accompanying textual explanations; and a personalized visual depiction of the interrelationship between a user's most recent CD4 count and viral load results. Some interview participants requested that information on the concept of Undetectable = Untransmittable (U = U) also be incorporated into the feature; this suggestion has since been implemented. The improved design elements from the Spanish PL iteration were subsequently incorporated into the English-language version of PL to improve usability of the laboratory results feature for English speakers who may have limited literacy or numeracy (Fig. 2).

## Discussion

Use of the PL m-health platform has been associated with increased viral suppression as well as decreases in stigma and increases in social support in majority English-speaking PLWH.^24–26^ However, PL was previously available only in English, likely excluding many Spanish-dominant PLWH from participating. Excluding limited English proficient patients from m-health creates unequal access to care services.^27^ Although Spanish speaking PLWH have shown reluctance to engage in m-health for HIV care,^28^ we found participants to be interested in m-health and willing to participate in the design of the application. Given the imperative to provide equitable health care services in a patient's preferred language, the PL team undertook this process of cultural adaptation, which yielded a tailored Spanish-language prototype and improvements in PL that have been extrapolated to other contexts. In this study, Spanish-speaking Latinx PLWH, who face many barriers to care, voiced a need for reliable information and social support, and they felt that a linguistically and culturally tailored adaption of the PL m-health intervention could help meet these needs.

Many of our participants entered this study with negative perceptions of technology related to prior experiences with inaccurate information and breaches in privacy. Maintaining privacy and confidentiality was the leading concern among participants, in accordance with prior m-health feasibility studies for stigmatized rural communities and PLWH.^29–32^ Providing secure access to curated Spanish-language educational materials in the app helps to address concerns regarding the availability of reliable information privately through technology. However, implementation of a Spanish-language PL will require acknowledging common and understandable concerns related to data security and privacy.

During the open-ended portion of interviews, participants did not initially express interest in using PL for social connection. After they tested the usability of the app, however, they stated that features providing social connection were the most motivating features for ongoing app usage. This finding underscores the need for best-practice user-centered design methods that began with research to get to know the participants' needs and contexts, was based on empathy, and used an iterative process that allowed for ongoing evaluation and improvement. Finally, it was important to seek preference data from users only after they have used a design and can tell how well it supports them.^33–37^ Social support can improve outcomes for PLWH^38,39^ and is a key feature of self-management of HIV disease.^40^ Because participants had limited prior experience with the use of technology for accessing social support to help cope with their HIV, it was unlikely to expect this feature to be sought out or requested by them. Originally, the concept for the community message board feature was driven by the literature on social support and its relevance to HIV management. Intervention development and adaptation can benefit from the use of both theoretical foundation and input from the priority population, used in a complementary iterative process that has been shown to meet user needs.^41–43^

Spanish-speaking Latinx PLWH in the nonurban Southern United States, where the density of Spanish speakers is low, may be particularly isolated due to cultural norms limiting disclosure to family and friends, not knowing other Spanish-speaking PLWH, inability to access support groups, and stigma.^6^ The low density of Spanish speakers at one nonurban site may limit the number of participants available to maintain discussion on a community board, indicating a potential need to pool participants from multiple sites. m-Health may provide an opportunity to create community and support regardless of geographic distance for Spanish-speaking PLWH, even though its possibilities were not independently identified by participants.

Ongoing feedback and a responsive development team are essential to ensuring success of an m-health platform. We highlighted an example where participant feedback led to significant changes in the laboratory results feature, improving participant understanding of their own health. This approach to adaptation of an m-health intervention emphasizes the importance of going beyond translation of English-language information. Although translation is necessary, it may not be sufficient to make an intervention accessible to groups not included in the original development.

Limitations of our study include the recruitment of participants from only two study sites, both in Virginia. The perceptions of our participants who live in areas with a low density of Spanish-speaking PLWH may not reflect those from more urban regions or other parts of the country. In addition, nearly all participants were born outside the continental United States, and, although representing numerous countries of origin, may have views that differ from Spanish-speaking Latinx PLWH born in the United States. Some of our participants reported English proficiency in addition to Spanish, and this may influence their perceptions regarding m-health and PL.

## Conclusion

Latinxs in the United States are disproportionately diagnosed with HIV and experience worse HIV-related health outcomes. Although m-health has demonstrated success in improving HIV care, a gap remains in the development of m-health platforms tailored to Latinx populations. Our formative study provides baseline attitudes about m-health among Latinx PLWH as well as desired m-health features. Privacy, confidentiality, and reliable information are key desired features. Social support through connection with other Spanish-speaking PLWH and with clinic staff provides a strong motivation for ongoing app use. m-Health interventions such as PL are acceptable to Spanish-speaking PLWH and involvement of the priority population in a user-centered formative process led to improvements in app accessibility and usability. Future research will include a postimplementation study regarding usability and acceptability of the Spanish-language adaptation for a cohort of Latinx PLWH.

## Supplementary Material

Supplemental data

## Abbreviations Used

CBO: community-based organization
PL: PositiveLinks
PLWH: people living with HIV
RWHAP: Ryan White HIV/AIDS Program
SD: standard deviation
U: U = undetectable equals untransmittable
