# Supplementary material for: Adapting an m-Health Intervention for Spanish-Speaking Latinx People Living with HIV in the Nonurban Southern United States
Source: Telemed Rep. 2021 Feb 3;2(1):46–55. doi: 10.1089/tmr.2020.0018 (PMC8009288; doi:10.1089/tmr.2020.0018)
Supplement: Supplemental data [file Supp_TableS1.docx]

**Supplemental Table 1**. Selected questions from semi-structured interview guide.

| Category | Sample Questions |
| --- | --- |
| Experience with mobile technology | - Do you have a mobile phone currently? If yes, continue with the following questions:   - What type of phone? (smartphone, mobile phone)   - What type of plan do you use? (pay as you go, phone contract)   - Do you share your phone with someone else?   - How often do you text? (once a day, more than once a day, weekly, less often)   - Do you use your phone to access the internet? How often?   - Is the language on your phone set to English or Spanish? If Spanish, which type of Spanish (Español, Español (Latinoamérica), Español (México), etc) - Do you have apps on your smartphone? What are your most used apps? What do you like about them? What do you not like about them? |
| Desired mHealth features | - Do you use any apps to help manage your HIV? What are they? What do you like about them? What do you not like about them - If you could design your very own app to help manage your HIV, how would it help you? What information or tools would you include in it? - In a smartphone application, what information or tools might be helpful when someone is first diagnosed, before seeing a medical provider? (More facts about HIV, help making appointments, a place to share your feelings?) - In an application, what information or tools would be helpful after starting to see a medical provider? (Reminders to take your medications or attend appointments, help making appointments) |
| Response to prototype app | Let’s start by talking your use of the application:   - Tell me about your general experience using the PositiveLinks application. - How motivated do you think you would feel to go into the app? (Do you think you would want to use the app or would you feel like you had to?) - What do you like most about the application? - Which features of the app do you think you would use most?   In term of specific features, do you think you would:   - Use the community message board?   - What would make you more/less likely to use the message board? - Look for information using the app? If so:   - Please show me where the Resources features is. What resources can you find in this section? What resources were missing? What is missing from this feature?   - Are you able to access the resources in Spanish? - Want to receive the daily questions about meds, mood, and stress? - Show me the dashboard feature on the app. How would you use this information? In what ways is this information helpful to you?   Spanish-language specific questions:   - Is the language and terminology in the application understandable? Are there any problems with translation? - What words, phrases or terminology used would you change to make better sense? Is there any wording that you found offensive? - In what ways is the application motivational? Do you think it would keep your interest? - In what ways are the materials culturally relevant? What suggestions do you have to improve it for other Spanish-speakers? |
| Response to prototype iteration | - What are your thoughts on the revised version of [specific app feature]? How could this feature be improved? |
